# ‘Us-Versus-Them’: Othering in COVID-19 public health behavior compliance

**DOI:** 10.1371/journal.pone.0261726

**Published:** 2022-01-24

**Authors:** Lauren Jodi Van Scoy, Bethany Snyder, Erin L. Miller, Olubukola Toyobo, Ashmita Grewal, Giang Ha, Sarah Gillespie, Megha Patel, Aleksandra E. Zgierska, Robert P. Lennon

**Affiliations:** 1 Department of Medicine, Penn State College of Medicine, Hershey, PA, United States of America; 2 Department of Humanities, Penn State College of Medicine, Hershey, PA, United States of America; 3 Department of Public Health Sciences, Penn State College of Medicine Hershey, Hershey, PA, United States of America; 4 Qualitative and Mixed Methods Core, Penn State College of Medicine, Hershey, Hershey, PA, United States of America; 5 Department of Family and Community Medicine, Penn State College of Medicine, Hershey, PA, United States of America; 6 Penn State College of Medicine, Hershey, PA, United States of America; 7 Department of Anesthesiology and Perioperative Medicine, Penn State College of Medicine, Hershey, PA, United States of America; 8 Penn State Law, Pennsylvania State University, University Park, PA, United States of America; Montclair State University, UNITED STATES

## Abstract

**Objective:**

We explored public perceptions about the COVID-19 pandemic to learn how those attitudes may affect compliance with health behaviors.

**Methods:**

Participants were Central Pennsylvania adults from diverse backgrounds purposively sampled (based on race, gender, educational attainment, and healthcare worker status) who responded to a mixed methods survey, completed between March 25–31, 2020. Four open-ended questions were analyzed, including: “What worries you most about the COVID-19 pandemic?” We applied a pragmatic, inductive coding process to conduct a qualitative, descriptive content analysis of responses.

**Results:**

Of the 5,948 respondents, 538 were sampled for this qualitative analysis. Participants were 58% female, 56% with ≥ bachelor’s degree, and 50% from minority racial backgrounds. Qualitative descriptive analysis revealed four themes related to respondents’ health and societal concerns: lack of faith in others; fears of illness or death; frustration at perceived slow societal response; and a desire for transparency in communicating local COVID-19 information. An “us-versus-them” subtext emerged; participants attributed non-compliance with COVID-19 behaviors to other groups, setting themselves apart from those Others.

**Conclusion:**

Our study uncovered Othering undertones in the context of the COVID-19 pandemic, occurring between groups of like-minded individuals with behavioral differences in ‘compliance’ versus ‘non-compliance’ with public health recommendations. Addressing the ‘us-versus-them’ mentality may be important for boosting compliance with recommended health behaviors.

## Introduction

The COVID-19 pandemic has drastically changed modern society and unearthed substantial societal flaws. As the pandemic continues, issues related to compliance with public health recommendations, including social distancing, vaccination, and masking have been hotly debated across the nation. The polarization of viewpoints and politicization of issues related to public health recommendations has become commonplace. Othering, which is described as a generic construct that may explain disparate public health outcomes based on perceived group identity within a society [1], has become apparent across the globe, with heightening misconceptions, xenophobia, and negative racial undertones particularly against those of Asian descent but also towards migrants, refugees, or other marginalized groups [2–6].

In March 2020, during the immediate period when the country was thrust into the era of social distancing [7,8], we conducted a mixed methods survey to evaluate public perceptions and intent to comply with early public health recommendations during the COVID-19 pandemic. During analysis of qualitative data related to public health behaviors, we were surprised by the pervasive political and social undertones that emerged in the qualitative, free-text responses to the survey, since our survey was conducted very early in the pandemic and was intentionally absent of political questions. The Centers for Disease Control and Prevention (CDC) recommendations at that time included social distancing, hand-washing, staying at home if feeling unwell, avoiding touching one’s face, and coughing or sneezing into the elbow. Masks were not yet recommended and vaccines were at the earliest stage of development.

Even though the survey was designed to focus on public health behavior, our initial qualitative analysis revealed that a new variant of othering was emerging even in the earliest days of the pandemic. Upon early analyses, it became clear that an ‘us-versus them’ undertone was pervasive throughout our data, reminisenct of, but dissimilar to, the common othering context of xenophobic and anti-Asian sentiments related to the origins of the COVID-19 pandemic [2–4]. The qualitative responses from our survey suggested that othering transcended racial or ethnic boundaries, and may also apply to the way in which individuals respond to public health recommendations of social distancing.

Accordingly, we conducted the present secondary analysis of the qualitative survey data to explore whether the ‘us-versus-them’ framing held true upon explicit examination. We present our findings using an othering lens, and to explore if and how othering is related to compliance with recommended health behaviors.

## Materials and methods

### Ethics statement

This study was approved by the Penn State College of Medicine Institutional Review Board (STUDY00014798) with Exempt Status. At the onset of the electronic survey, participants were provided a Summary Explanation of Research. All participants provided implied inform consent in accordance with IRB regulations.

### Survey design

We used a convergent mixed-methods approach to design a cross-sectional survey to investigate the following constructs: public COVID-19 knowledge; intent to follow CDC recommendations; perceptions about COVID-19; and preferred information sources [8].

Four qualitative (open-ended free text) questions were included in the survey: 1) *What worries you most about the COVID-19 pandemic*?; 2) *What*, *if anything*, *prevents you from following CDC recommendations about COVID-19*?; 3) *How do you feel about the way information regarding COVID-19 has been delivered to you*?; and 4) *Is there anything else you would like to share regarding the COVID-19 pandemic*?

Since no validated COVID-19 quantitative questionnaires existed when we designed the study, quantitative questions were modeled on the validated European “Standard questionnaire on risk perception of an infectious disease outbreak” [9]. To assess knowledge, questions were written based on published information from the CDC website [10]. Next, the survey was refined through two rounds of cognitive interviewing procedures with 13 individuals utilizing the ‘think-aloud’ technique [11]. Finally, pilot testing with a random sample of 1,000 potential participants was performed to ensure adequate knowledge discrimination and qualitative sensibility. Questions were revised as appropriate.

The final mixed methods survey included 65–92 items, depending upon branching logic applied to individual answers. The survey assessed four constructs: 1) knowledge and confidence in that knowledge (15 items for knowledge; 15 items for confidence); 2) intention to follow and beliefs about CDC recommendations (10 items); 3) perceptions and concerns about COVID-19 and other infections (15 items); 4) information sources (7 items); and 5) demographic questions (18 items) [8]. The four qualitative questions that were included in the survey were designed to align with these quantitative questions, based on the initial research questions [8].

The survey was administered online from March 25–31, 2020, in the days immediately following the federal COVID-19 ‘lockdown’ order. The online REDCap [12] survey was sent to a convenience sample of 121,573 adults from a healthcare system in Central Pennsylvania. For the quantitative analysis, participants were included if they were adults who agreed to participate in the health center’s marketing directed listserv and also agreed to complete the electronic survey.

**Fig 1:**
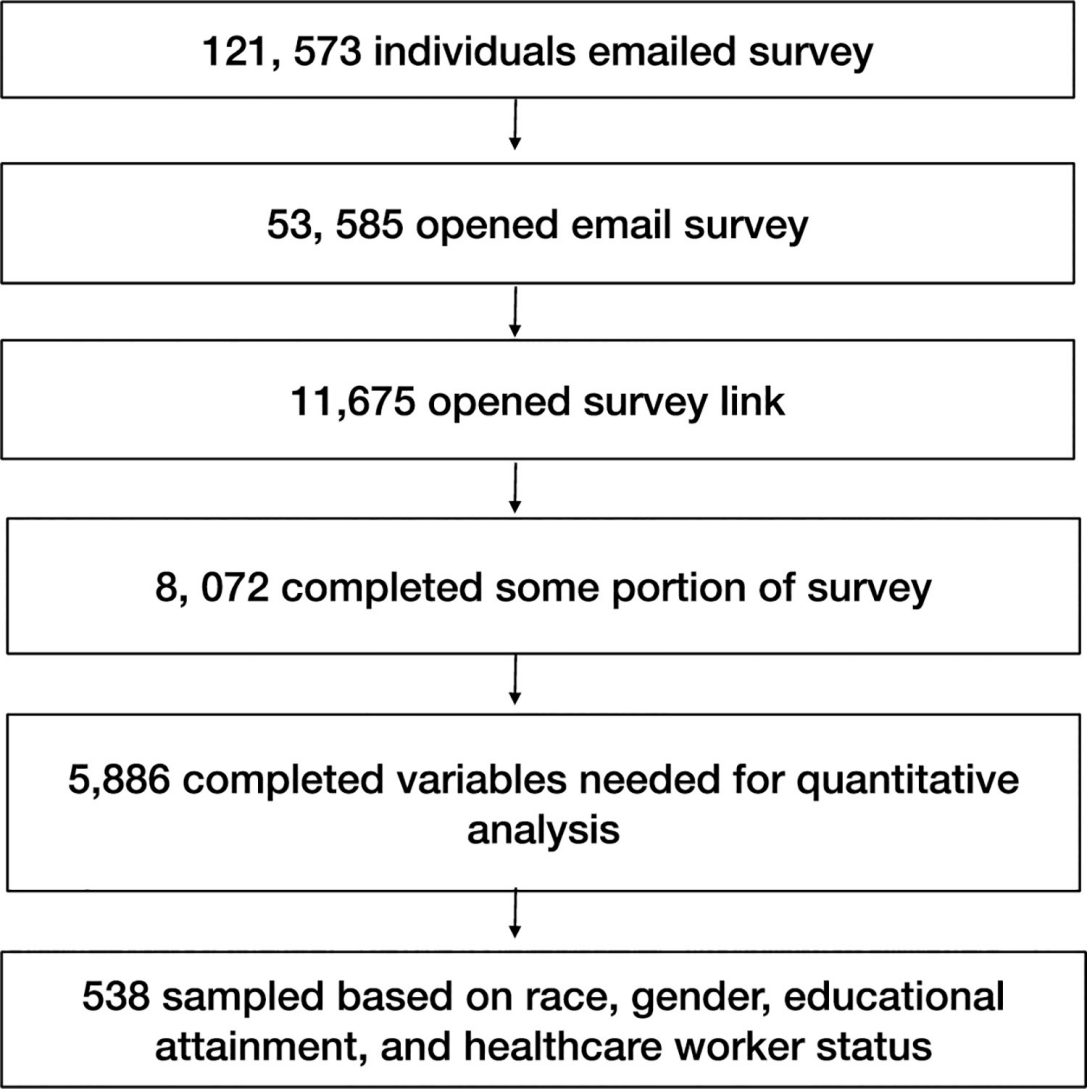
Participant sampling procedure.

For the qualitative analysis reported here, we used stratified purposive sampling to select 538 of the 5,948 (approximately 10% of respondents) for qualitative analysis based on education status, race, gender, and healthcare worker status (**Fig 1**) [13]. These strata were chosen because quantitative analyses indicated that these variables revealed differences in interpretation of COVID-19 related content [14]. Further, race is an important construct when considering othering phenomena, since othering is typically described with regards to racial minority groupings, we sampled to capture diversity of both race. Educational status and healthcare worker status were variables chosen for stratification because it is reasonable to expect that differences in these areas may translate to how one perceives or interprets health related information related to COVID-19. Thus, sampling was performed to capture variability within those potentially different perspectives.

### Qualitative analysis

We used an ontological philosophical assumption when analyzing the data. This perspective is appropriate when seeking the nature of ‘reality’ (in this case, how and why individuals describe ‘us-versus-them’ in a COVID-19 related context) [15,16]. We applied a pragmatic, inductive coding process to conduct a qualitative, descriptive content analysis [17,18]. This approach prioritizes the face value of the content to provide an accurate description of the phenomenon.

This manuscript adheres to the Consolidated Criteria for Reporting Qualitative Research (COREQ) guidelines [19]. The lead qualitative researcher (LJV) is a physician scientist with a research interest in communication, decision-making, and behavioral science. Her experiences with medicine and research give perspective into the meaning behind how health recommendations may affect behavior and decision-making. She has a background in medical humanities and philosophically believes that individuals make meaning through their experiences and choose behavior based on emotions as well as rational problem-solving skills.

The lead qualitative researcher led the development process for a preliminary codebook that was created by using descriptive categories and concepts that emerged from the survey responses. The data was analyzed as a whole (inclusive of all four qualitative responses) as opposed to question by question since the ‘us-versus-them’ pattern emerged across questions. This preliminary codebook was created using an early analysis of 250 different surveys from the same dataset [8]. An additional 250 surveys (each with 4 qualitative responses) from the new purposive sample of 538 surveys were examined and used to refine the initial codebook by deepening the description and specificity of the codes. Then, six analysts were trained to use the constant comparison method to code 75 surveys (2 coders per response) using this codebook [20]. Coding discrepancies were reconciled through discussion over the course of three team meetings. When intra-class coefficients (‘kappa’) were 0.65 among all 6 coders, the remaining 425 responses were coded (2 coders per response). NVivo Version 12 software was utilized to assist with qualitative data coding, organization, and visualization of patterns within the data.

Coding patterns were then reviewed within each category; analysts bracketed biases by grounding the analysis in verbatim quotes to maintain neutrality [21,22]. Four major patterns of coding emerged: health concerns, societal concerns, distrust, and media messaging concerns. The size and depth of each category required separate treatment of each. This manuscript reports on codes related to othering; distrust and media messaging concerns are reported separately [8,23]. The ‘us-versus them’ framework was applied to the data after the initial coding and preliminary analysis. In other words, the researchers did not ‘look’ for ‘us-versus-them’ but rather reviewed the data using this lens after the framework emerged as relevant during the inductive process.

### Subgroup analysis

To learn if the strata had an influence on the findings, the crosstab matrix from NVivo 12 software was used to organize data into the strata groups: race, gender, educational attainment, and healthcare worker status [24]. These subgroups were chosen because they showed differences in COVID-19 related knowledge on the quantitative analysis from the survey [8]. Coding patterns, frequencies and groupings for each category were examined in the subgroup matrix (**S1 Table**) and frequencies and row percentages were calculated by NVivo software. Within each cell, descriptive content and categories were examined and compared using the constant comparison method [20].

### Quantitative and mixed methods analyses

Descriptive statistics were used to compile means, standard deviations, frequencies, and percentages from demographic survey items. We used a narrative approach to integrate the quantitative (demographic) data with qualitative data [25].

## Results

### Participants

Among the 538 purposively sampled survey respondents (Table 1), 50% identified as racial minorities and 58% as female, 56% had at least a bachelor’s degree, and the mean age was 53.0 (SD 17.8) years. Characteristics of both the full sample and the purposive sample are shown in Table 1. There were no meaningful differences in adherence with public health behaviors between the full and purposive samples (**S2 Table**).

**Table 1:** Participant demographics.

Variable	Percentage of Full Survey Sample N (%) or Mean (SD); n = 5,886	Percentage of Qualitative Purposive Sample, N (%) or Mean (SD); n = 538)
***Age*, *Years***		
Mean (SD)	56.4 (1.9)	53.0 (17.8)
***Gender***		
Male	1862 (31.6)	217 (40.3)
Female	3961 (67.3)	313 (58.3)
Non-binary/Prefer not to Answer	41 (0.7)	2 (0.4)
Missing	23 (0.4)	6 (1.1)
***Race/Ethnicity***		
American Indian or Alaska Native	19 (0.3)	19 (3.5)
Asian	56 (1.0)	57 (10.6)
Black or African American	98 (1.7)	95 (17.7)
Hispanic or Latino	91 (1.5)	92 (17.1)
Native Hawaiian or Other Pacific Islander	3 (0.1)	3 (0.6)
White	5411 (91.9)	266 (50.5)
Missing	22 (0.4)	6 (1.1)
***Highest Level of Educational Attainment***		
Did Not Finish High School	36 (0.6)	26 (4.8)
High School	726 (12.3)	41 (7.6)
Some College	963 (16.4)	99 (18.4)
Associate’s Degree	651 (11.1)	71 (13.2)
Bachelor’s Degree	1670 (28.4)	178 (33.1)
Graduate Degree	1823 (31.0)	123 (22.9)
Missing	18 (0.3)	0 (0)
***Do you work in the medical profession*?**		
Yes	933 (15.9)	146 (27.2)
No	4908 (83.4)	390 (72.8)
Missing	46 (0.8)	2 (0.4)

### Themes

Ten themes emerged from the overall qualitative dataset. Four themes related to health, societal, and behavioral concerns are presented in this manuscript; the remaining six themes related to messaging, distrust, and economic concerns are reported elsewhere [8].

We analyzed the findings using an othering lens to better contextualize ‘us- versus-them’ undertones that emerged as a common concept linking the four presented health and society themes. Othering is a construct that creates boundaries of ‘us-versus-them’ and has implications for health outcomes based on power relations. Othering has been used to understand health inequities in marginalized (othered) groups as well as other social contexts [1]. Although our study was not originally conceptualized using the othering framework, the findings resulted in an analysis that involved contextualization of results through the othering lens because participants attributed negative COVID-19 behaviors to other groups, setting them apart as representing characteristics opposite to the participants.

#### Theme 1. Participants expressed widespread lack of faith in others and expressed doubt that others would comply with public health recommendations

While the vast majority expressed few, if any, barriers to their personal ability to adhere to public health recommendations, such as hand washing or social distancing, there was extensive concern about the perceived lack of concern others exhibited in following pandemic-related preventive health measures. Participants described concerns that ‘others’ were not listening to recommendations such as staying at home or were dismissive and cavalier about the pandemic.

*“What worries me the most is that there are* ***so many people*** *who are still going about their regular lives*, *with little or no caution regarding the spread of the virus*.*” –*39 year old female, American Indian, Bachelor’s degree, non-healthcare worker

Participants connected these behaviors of ‘others’ as the cause of ongoing spread of the virus.

*“****Other people*** *are not staying home*. ***I’ve*** *stayed home for 11 days now*. *If/when I venture out after my 14 days*, ***they*** *could still be spreading it around*.*”* –50 year old female, American Indian, Graduate degree, non-healthcare worker

Frequent expressions of anger and frustration toward others were notable in the responses, often with perceptions about others being uncaring or self-centered.

*“****Young adults*** *believe they are invincible and do not consider* ***other people’s*** *need*.*”* 70 year old female, white, Bachelor’s degree, non-healthcare worker

*“I worry that our nation has become so selfish and narcissistic that we cannot see the greater good*.*”* 40 year old female, Asian, Bachelor’s degree, non-healthcare worker

#### Theme 2. Participants’ fears of illness or death were pervasive and encompassed issues pertaining to both *their* inner circle as well as *other* at-risks groups

Participants were concerned with personal illness, although this theme was less prevalent than the concern about ‘others’ (Theme 1). Concerns for oneself or family were most commonly tied to risk conditions, such as age, immunocompromised states, or other chronic illnesses.

*“****Our son has adrenal insufficiency*** *from his cancer treatment*, *and* ***I have asthma***, *so my biggest fear* ***is anyone in our family*** *getting the virus*.*” –*47 year old female, white, Bachelor’s degree, non-healthcare worker

*“****I am 76 years old with heart and lung issues*** *and meet all of the standards for a bad outcome from the virus*.*” –*76 year old male, white, Bachelor’s degree, non-healthcare worker

A subtheme included worries about illness in general, not only COVID-19-related illness. Specifically, the participants were concerned about lack of access to non-COVID-related healthcare, including mental health, now or in the future.

*“****I’m affected in a different way***, *I was scheduled for spinal surgery*, *however it has been cancelled indefinitely until things get under control*. *I’m living in excruciating pain every day with no end in sight…” –*44 year old female, white, Associate’s degree, healthcare worker

*“****I am pregnant*** *and* ***worried about my baby***. *I don’t see any information on the effect of COVID 19 on new born and how delivery process will be*? *What happens if C-section is needed during this time*?*” –*35 year old female, white, Graduate degree, non-healthcare worker

There was approximately equal representation of concerns for oneself as for ‘others’ from at-risk groups, such as healthcare workers, the elderly, infirm, or those with mental illness. Healthcare workers themselves were especially concerned with spreading the virus to family members.

*“****I worry about the doctors*, *nurses and EMTs*** *more than myself or my family*. *If they get sick*, *who’s going to treat them*?*” –*60 year old female, white, Associate’s degree, healthcare worker

*“****I am also concerned about the mental health of all***, *particularly those who are already diagnosed with mental illness*.*” –*62 year old female, white, Associate’s degree, healthcare worker

*“****I am most concerned with being immunocompromised*** *and possibly bringing this virus home to not only my husband but also my 19-month old son*. *It concerns me that I am now the biggest threat to my family*.*” –*27 year old female, white, Associate’s degree, healthcare worker

#### Theme 3. Participants expressed frustration at perceived slow societal response to the pandemic and perceived lack of standardized plan for the country

We previously reported substantial distrust in the executive branch of government related to messaging and information dissemination [26]. Separate from this distrust was a strong dissatisfaction with the governmental response to the COVID-19 pandemic. Participants perceived the national response as inadequate or too slow, and that this response indicated a lack of preparation that worsened the impact of the pandemic.

*“It is extremely worrisome that our country has dropped the ball on prevention of this virus*. *If we were more prepared to prevent this*, *we would not have to be as reactive as we are now*.*” –*40 year old female, white, Associate’s degree, non-healthcare worker

*“If we were educated as to the possibility of this pandemic hitting our country*, *the citizens could have been better prepared and systems set up in the event that work places had to shut*. *I am shocked that this was not assessed prior by our government*.*” –*70 year old female, white, some college, healthcare worker

Participants also expressed frustration at the perceived lack of a coordinated national response.

*“The fact that every state in the US has done something different to prevent the virus does not make much sense*.*” –*29 year old male, white, Bachelor’s degree, non-healthcare worker

Finally, participants expressed concern over seemingly inadequate resources (e.g., personal protective equipment, hospital beds, ventilators) to deal with the pandemic, thereby contributing to anxiety, frustration, and distrust of the governmental response.

*“Knowing that there is not enough PPE equipment for health care providers and ventilators for patients is terrifying*.*” –*62 year old female, white, some college, healthcare worker

*“Lack of testing all lead me to believe CDC is playing games or doesn’t care*.*” –*54 year old male, Hispanic, Bachelor’s degree, non-healthcare worker

*“Poor leadership at federal government level is sacrificing public health in favor of economic aims*. *This has already handicapped the response to the pandemic*.*” –*68 year old male, white, Bachelor’s degree, non-healthcare worker

#### Theme 4. To overcome concern about unknowns of the virus itself, participants desired a data-driven approach to guide the pandemic response, including transparency of local COVID-19 statistics

Participants had a variety of concerns about viral behavior, including prevalence, incubation, and immunity.

*“I want to know more about how fast it spreads*, *how long exactly the incubation period is*, *all the non-typical symptoms*.*” –*41 year old female, white, Graduate degree, non-healthcare worker

*“The mannerism of the virus*. *The length of time it remains on different surfaces*. *Is there a possible chance someone can have the virus for a second time*?*” –*56 year old female, white, did not finish high school, non-healthcare worker

The most common concern, however, was asymptomatic viral spread. This concern was most closely linked with participants’ desire to have detailed data about real-time, local case rates and how close the virus was to their own community or home.

*“When local cases were identified*, *I would have liked to know details about person’s locale*, *where possible exposure happened*, *were they travelling*, *age range*, *did they have underlying health issues (while protecting person’s personal info)*.*” –*63 year old male, white, Bachelor’s degree, non-healthcare worker

While many acknowledged the need to protect patient privacy, the desire for such specific, potentially identifiable information suggested that some prioritized the value of local statistics and information over others’ individual confidentiality.

*“Need to hear the breakdown of ages more completely*, *how sick the patient is or was*. *The state of PA will not give you specifics as to where in a county the infected are*. *I think that the privacy is important*, *but the societal health concerns outweigh the confidentiality*.*” –*69 year old male, white, Graduate degree, non-healthcare worker

### Subgroup analyses

Subgroup analyses were conducted to explore differences in theme codes based on gender, race, educational attainment, and healthcare worker status. Codes were grouped into categories for the purpose of this analysis (**S3 Table**). Codes were not mutually exclusive to each category. Categories were analyzed separately. Differences of greater than 10% between coding frequency and subgroup population percent representation were considered potentially meaningful (**Table** 2).

**Table 2:** Subgroup analysis showing frequencies of coding per subgroup across categories (N = 532)^a^.

Subgroup	Percentage of Sample, N (%)	Coding Categories^a^ n (%)					
		**Health Concerns for Self or Others n (%)**	**Lack of Concern of Others n (%)**	**Economic Concerns n (%)**	**Lack of Societal Preparedness n (%)**	**Viral Behavior n (%)**	**Vaccine/meds n (%)**
***Race***							
White	**266 (50.0)**	160 (54.8)	68 (59.1)	42 (60.0)	81 (49.7)	97 (49.5)	39 (47.0)
American Indian or Alaska Native	**19 (3.6)**	11 (3.8)	8 (7.0)	1 (1.4)	5 (3.1)	7 (3.6)	2 (2.4)
Asian	**57 (10.7)**	28 (9.6)	10 (8.7)	10 (14.3)	21 (12.9)	30 (15.3)	13 (15.7)
Black	**95 (17.8)**	42 (14.4)	14 (12.2)	8 (11.4)	29 (17.8)	31 (15.8)	15 (18.1)
Hispanic	**92 (17.3)**	50 (17.1)	14 (12.2)	9 (12.9)	26 (16.0)	30 (15.3)	14 (16.9)
Native American	**3 (0.6)**	1 (0)	1 (0.9)	0 (0.0)	1 (0.6)	1 (0.5)	0 (0.0)
All Races^b^	**532 (100.0)**	292 (54.9)	115 (21.6)	70 (13.2)	163 (30.6)	196 (36.8)	83 (15.6)
***Biological Sex***							
Female	**313 (58.8)**	184 (58.8)	73 (63.5)	43 (61.4)	87 (53.4)	120 (61.2)	46 (55.4)
Male	**217 (40.8)**	107 (49.3)	42 (36.5)	27 (38.6)	76 (46.6)	76 (38.8)	37 (44.6)
Other	**2 (0.0)**	1 (0.3)	0 (0.0)	0 (0.0)	0 (0.0)	0 (0.0)	0 (0.0)
All Biological Sexes^b^	**532 (100.0)**	292 (54.9)	115 (21.6)	70 (13.2)	163 (30.6)	196 (36.8)	83 (15.6)
***Educational Status***							
< Bachelor’s Degree	**237 (44.5)**	134 (45.9)	55 (47.8)	**19 (27.1)**	**56 (34.4)**	77 (39.3)	31(37.3)
≥ Bachelor’s Degree	**295 (55.5)**	158 (54.1)	60 (52.2)	**51 (72.9)**	**107 (65.6)**	119 (60.7)	52 (62.7)
All Educational Statuses^b^	**532 (100.0)**	292 (54.9)	115 (21.6)	70 (13.2)	163 (30.6)	196 (36.8)	83 (15.6)
***Healthcare Worker Status (2 missing data*, *n = 530)***							
Non-Healthcare Worker	**385 (72.6)**	91 (68.6)	73 (64.0)	50 (71.4)	121 (74.7)	142 (72.8)	62 (75.6)
Healthcare Worker	**145 (27.4)**	199 (31.4)	41 (36.0)	20 (28.6)	41 (25.3)	53 (27.2)	20 (24.4)
All Worker Status^b^	**530 (100.0)**	290 (54.7)	114 (21.5)	70 (13.2)	162 (30.6)	195 (36.8)	82 (15.5)

Percentages are row percent, such that of the coding for each topic, the percentage indicates the percentage of those coding for the topic were categorized in each subgroup class.

Bolded values represent a difference in coding frequency of >10% base on sample size representation. For example, 72.9% of those with ≥Bachelor’s degree expressed economic concerns but represent only 55.5% of the sample.

^a^ 6 participant surveys were excluded due to missing responses.

^b^ Codes were collapsed into categories for purposes of reporting subgroup patterns; these categories are not mutually exclusive.

The only between-subgroup differences meeting this threshold were seen based on educational attainment. Those with Bachelor’s degree or higher more commonly expressed concerns about the economy than would be expected based upon the sample distribution of those with Bachelor’s degree or higher (55.5%). Similarly, those with less than Bachelor’s degree less commonly expressed concerns about the economy (27.1%) which is less than expected based on the sample distribution (44.5%) using the cutoff of 10%. Differences were also seen in coding for lack of societal preparedness: 65.6% for those with higher than a bachelor’s degree compared to the 55.5% sample distribution, and 34.4% for those with less than a bachelor’s degree compared to the 44.5% sample distribution). There were no potentially meaningful differences seen by race, gender or healthcare worker status across the coding categories.

For all subgroup analyses described above, examination of the *descriptive* content of each category by subgroup (using the constant comparison method) did not reveal any obvious differences with regards to subgroups. In other words, the *way* the content was expressed was not qualitatively different across subgroups.

## Discussion

To our knowledge, this is the first large, rigorous, qualitative analysis of public perceptions about, and intent to comply with, public health recommendations related to the COVID-19 pandemic in a sample of adults from diverse backgrounds. We previously published quantitative findings reporting that only 67% of surveyed participants intended to comply with social distancing and travel restrictions at the time [7,8]. A robust literature is emerging in peer-reviewed and pre-print manuscripts that quantitatively links fear and anxiety to intent to comply with behaviors [27–29]. Themes from our qualitative analysis of that dataset explored some of the deep-seated concerns of survey participants. We uncovered a variety of concerns, including a widespread lack of faith that others will follow public health recommendations, fear for both one’s inner circle and other at-risk groups, desire for detailed reporting of local COVID-19 cases to overcome fears of asymptomatic spread, and frustration at both a perceived lack of preparation and slow federal response to the pandemic.

Linking all of these themes was a subtext related to Othering undertones [30]. This was demonstrated in Theme 1 through sentiments from participants that while ‘I’ am doing what needs to be done, ‘they’ are not, and that is putting all of ‘us’ at risk. In Theme 2, participants described concerns about illness with regards to their inner circle of self, family and friends (‘us’) as well as societal ‘at-risk groups,’ such as healthcare workers, or those with illnesses (‘them’). There was ‘us-versus-them’ in Theme 3 through expressions of substantial distrust in the way ‘they’ (government and media) had responded inadequately or too slowly to the pandemic, putting ‘us’ all at risk. Even sentiments from Theme 4, which focused on strategies participants stated would help overcome their concerns about viral behavior, included undertones of ‘them’ as ‘the carriers,’ and that local statistics and detailed demographic data identifying the ‘others’ were needed to protect oneself and family (‘us’). Further, we found qualitative similarities across race and gender groups, with differences in coding patterns noted only based on educational attainment. It is important to note that additional themes, including governmental distrust and media sensationalism emerged from the data as well, and supported the ‘us-versus-them’ construct discussed here, however, due to the complexity and richness of this large qualitative dataset, those themes are considered separately and published elsewhere.

Many believe the COVID-19 pandemic has highlighted the politicization of healthcare in the US and further divided the nation [31]. Our data support this notion suggesting that othering–a process that ‘identifies those thought to be different from oneself or the mainstream [30]–is a common lens through which patients express their concerns about the pandemic. Othering has been described in the context of healthcare service disparities and discrimination against a variety of groups in other healthcare contexts, including among minority groups, drug users, or who identify as non-binary [30,32,33]. Othering is gaining recognition as an explanation for disparities in public health [1].

Notably, we identified Othering as occurring not between racial or ethnic groups, but rather between groups of like-minded individuals with behavioral differences–‘compliance’ or ‘non-compliance’ with public health recommendations. Thus, in the COVID-19 era, our data suggest an emergence of a new consideration of acceptance or non-acceptance of social norms as they relate to public health recommendations. The implications of this new class of Othering are troubling as Othering serves to normalize and justify different treatment for different groups, typically as a hierarchical power relationship, in western cultures archetypically based on racial or gender divides [5,34]. As overcoming health disparities is a primary goal in US healthcare, identifying a new source of Othering is problematic, particularly in the context of COVID-19-related othering, as othering among vaccinated and non-vaccinated is closely linked with political division in the United States [35]. Politicizing healthcare does not help patients [36], and politicizing pandemics promotes othering in the forms of ethnic and racial discrimination, hate speech, and aggression [37].

Limitations include data collection from a single state and single health system, early during the pandemic, which may limit generalizability to other regions and other stages of the pandemic, as COVID-19 events evolve. Further, we did not examine cultural or political attitudes and affiliations, variables that are likely to impact perceptions about the COVID-19 pandemic. That said, this was intentional because the research was designed originally to avoid triggering political bias. Further, as a voluntary survey, there is potential for self-selection bias in that participants who did not complete the survey may have different views and perspectives from those who completed it. Finally, individuals who are not part of the health system were not surveyed.

Our study has several strengths. We adhered to published guidelines of qualitative rigor, used purposive sampling to include a diverse sample, and included a large sample size to ensure data saturation within all subgroups.

In summary, many adults perceive COVID-19 through an ‘us-versus-them’ lens, reminiscent of othering. Participants used dichotomous terms to confine the problems to the others based on compliance or non-compliance with public health recommendations, fears of illness or death, government response, and even expressing solutions to their concerns. Health policy historically relies on interventions targeting health outcomes defined by social determinants of health, like race and ethnicity. Thus, our findings are relevant for public health researchers and policy-makers, who are facing an unprecedented healthcare challenge from disparate health practices and outcomes and othering in a divided nation. An important consideration for policy-makers who are responding to the COVID-19 pandemic is to address the ‘us-versus-them’ mentality of compliers versus non-compliers to strengthen the impact of public health messaging campaigns and improve compliance with recommended health behaviors.

## Supporting information

S1 Table Final codebook and frequency of coding. (DOCX)

S2 Table Adherence with health recommendations. (DOCX)

S3 Table Categories used in subgroup analysis. (DOCX)
