# Supplementary material for: ‘Us-Versus-Them’: Othering in COVID-19 public health behavior compliance
Source: PLoS One. 2022 Jan 24;17(1):e0261726. doi: 10.1371/journal.pone.0261726 (PMC8786185; doi:10.1371/journal.pone.0261726)
Supplement: S1 Table — (DOCX) [file pone.0261726.s001.docx]

| Table S1. Final Codebook and Frequency of Coding | | |
| --- | --- | --- |
| Category* and Code | Description | References |
| **1. Distrust** | | |
| Of Other Countries; Conspiracy theories | Participant reports not trusting information/data reported from other countries. Conspiracy theories. | 8 |
| Of Other Entities (not president) | Participant believes governmental leaders do not heed medical advice of experts. | 18 |
| Of President and his administration | Participant reports distrust in the US government (distrust in President/his administration; executive branch specifically) | 16 |
| Concern President is influencing CDC or Scientists | Participant reports concern that the president is influencing, or playing a role in CDC and/or scientists filtering or editing their messaging to the public. Reports presidents does not allow medical experts to have a voice. | 32 |
| Distrust in president's mixed, false  messages | Participant reports distrust in the president's inconsistent, mixed or false information. | 105 |
| Leaders not listening to medical advice | Participant reports government leaders do not listen to expert medical advice such as Dr. Fauci/CDC. | 50 |
| Politicization | Participant is concerned that politics/the upcoming election have played a role in decisions or information. | 56 |
| Other distrust | Participant is concerned about other issues related to government | 47 |
| **2. Media Messaging** | | |
| Confusing, Inconsistent, Mixed messages | Participant reports that COVID messaging is confusing, mixed and/or inconsistent. | 119 |
| Media worsening panic; Scary; Sensationalized | Participant believes the media is creating a hype/panic within the public. Messaging is reported as scary or sensationalized. | 87 |
| Over-information from sources other than the media | Participant reports receiving too much information, feeling bombarded by information sources that are not the media. | 51 |
| Other Media Messaging | Any other references to media messaging that were not described in the above codes. | 56 |
| **3. Trusted Sources of Information** | | |
| CDC or WHO | Participant believes the CDC or WHO is a trusted source of information. | 59 |
| Daily Press Updates | Participant reports trusting daily updates by the president or state governor, Dr. Levine, etc. | 31 |
| I don't know who to trust | Participant reports not knowing what information source to trust. | 2 |
| Prefer state info, Governor, Levine | Participant believes that state government/governor/Dr. Levine (physician general) are more reliable than federal government. | 49 |
| Scientists | Participant believes scientists and experts are trusted sources of information. | 6 |
| TV News Networks | CNN, MSNBC, etc. | 103 |
| Other Trust Sources of Info | Any other trusted sources of information that were not described in above codes. | 154 |
| **4. Personal Medical Concerns** | | |
| Death of self | Participant is concerned about dying from COVID. | 66 |
| Fear of getting sick | Participant reports fear of contracting COVID-19 and becoming sick. | 194 |
| Inadequate work policies | Participant explains concern that workplace policies do not protect them in exposure to patient/at-risk population. They are classified as an 'essential worker' but do not believe they are truly that designation. | 20 |
| Questions or Concerns about medication; drug development, treatment | Participant concerned or has questions about pharmaceutical drug development for COVID, treatments for COVID, etc. Delay in treatment and/or diagnosis. | 139 |
| Questions or Concerns about personal access to care or testing (self or family) | Participant worries about lack of access to COVID testing or the delay in testing for themselves or their family. Concern about lack of access to care related to COVID. | 31 |
| Other Personal Medical Concerns | Participant is worried about other medical issues not mentioned | 44 |
| **5. Family Concerns** | | |
| Family member dying | Participant reports concerns of a family member and/or loved one dying as a result of COVID-19. | 43 |
| Family member illness | Participant is worried about their loved one’s compromised immune system or other underlying risk factors (e.g. older age, diabetes, heart disease). Concerned about a family member dying from COVID. | 196 |
| Not being able to provide care for family if sick | Participant worries that they will not be able to provide care for a family member if the participant themselves were to get sick. | 5 |
| Other Family Concerns | Any other family concerns that were not described in above codes | 29 |
| **6. Societal Concerns** | | |
| **6A. Economic** |  | **0** |
| Affecting at-risk populations (elderly, ill, etc.) | Participant worries that at-risk populations will be disproportionally affected. | 0 |
| Economy crashing | Participant is concerned about an economic crash/fall out/decline. | 70 |
| Hoarding, Scarcity of Essential Items | Participant is worried about shortages in food supplies, everyday supplies, household items, etc. | 25 |
| Job layoffs | Participant is concerned about losing their jobs and/or others losing their jobs. | 31 |
| Lack of societal, public health preparedness | Participant worries that society lacks the infrastructure to be prepared for a pandemic. E.g. We are under-prepared and under-resourced and lack political leadership. It is extremely hard on the medical workforce who have to try to minimize the fallout, not to mention the population at large, who have to deal not only with illness and possible bereavement, but also job losses and all the other consequences of a ruined economy. | 174 |
| Other Economic Concerns | Any other economic concerns that were not described in the above codes. | 52 |
| **6B. Epidemiologic** | | |
| Concern of Racial or Socioeconomic Injustices, Health Inequity | Participants mentions concerns of racial issues, marginalization, unequal access to healthcare resources/access. Concerned about living necessities (food, medications, etc.) shortages particularly for underserved groups. | 14 |
| Lack of concern of others | Participant worries that other’s actions show a lack of concern about the seriousness of the pandemic | 245 |
| Questions or Concerns about Lack of vaccine | Participant is concerned about a lack of a vaccine to for COVID. | 87 |
| Questions or Concerns about Overwhelm of healthcare resources (staff, PPE) | Participant is concerned for healthcare workforce shortages, such as doctors, nurses, medical assistants, etc., shortages of healthcare supplies such as PPE, ventilators, and/or lack of healthcare facilities | 149 |
| Questions or Concerns about Testing | Participant worries about reported false positive cases. | 124 |
| Questions or Concerns about Viral Behavior | Participant wishes to know more about asymptomatic transmission rates, prevalence, disease process, severity, mutation, symptom identification of COVID | 460 |
| Other Epidemiologic Concerns | Any other epidemiologic concerns that were not described in the above codes. | 48 |
| **6C. Safety and Health of Others** | | |
| At-Risk populations (not personal family) | Participant concerned about health at-risk populations (e.g. immunosuppressed, heart disease, liver disease, diabetes, elderly, etc.) | 53 |
| Concern about Mental Health | Participant concerned about mental health ramifications of social isolation/quarantine. | 10 |
| General loss of life, death (societal level) | Participant is concerned about mass loss of life. | 69 |
| Healthcare Workers | Participant reports concern for health and safety of healthcare workers, the healthcare workforce, etc. | 51 |
| Other Safety and Health Concerns | Any other concern for safety and health of others not described in the above codes. | 58 |
| **6D. Panic** | | |
| False fear, panic, paranoia | Participant is worried about the public fear, panic and paranoia | 84 |
| Hoarding | Participant worries about the public hoarding | 20 |
| Lack of normality | Participant’s life lacks normal routines and/or consistency. | 52 |
| Overreaction | Participant reports that the public’s, media’s and/or government’s response to the COVID-19 pandemic is an overreaction. References to the pandemic isn’t as serious as its being made out to be. | 14 |
| Uncertainty, the unknown | Participant is worried about current state of uncertainty and/or an uncertain future. This node should have an existential interpretation. | 57 |
| Other Panic | Any other panic not described in the above codes. | 17 |
| 7**. Barriers to Recommendations** | | |
| Forgets the recommendations | Participant does not follow CDC recommendations because they forget what they are. | 4 |
| Hard to change habits | Participant reports not following CDC recommendations due to their own personal habits | 20 |
| Lack of access to sanitizer | Participant not having enough sanitizer to adhere to guidelines. | 2 |
| Legal obligation to travel (e.g. child visitation) | Participant is legally required to travel (e.g. travel for child visitation, etc.) | 1 |
| Loneliness and mental health | Participant reports not following all CDC recommendations due to addressing their own mental Participant health needs and/or their own loneliness. | 11 |
| Must provide care or support to family | Participant leaves their home to provide care for and/or support a family member. | 19 |
| Need to buy essentials | Participant reports violating CDC recommendations due to the needs to leave their home to buy essentials. | 93 |
| Need to get healthcare | Participant reports leaving their home to receive necessary healthcare. | 14 |
| No barriers | Participant reports experiencing no barriers to following CDC recommendations. | 483 |
| Small business | Participant reports not following CDC recommendations because they run a small business. | 1 |
| Thinks overkill, over-reaction | Participant does not adhere to the CDC recommendations because they believe aggressive and unnecessary/’overkill.’ This is different than “over-reaction” which is in general and not tied to behavior. | 8 |
| Work requires interactions or travel | Participant’s work requires them to interact in close quarters with others and/or their work requires them to travel | 99 |
| Other Barriers to Recommendations | Any other barriers to recommendations not described in above codes. | 59 |
| **8. No worries** | Participant reports not being worried about messaging related to COVID. | 33 |
| **9. Other, Broad** | Any other response that does not in into any other categories or codes above. | 34 |
