# Supplementary material for: ‘Us-Versus-Them’: Othering in COVID-19 public health behavior compliance
Source: PLoS One. 2022 Jan 24;17(1):e0261726. doi: 10.1371/journal.pone.0261726 (PMC8786185; doi:10.1371/journal.pone.0261726)
Supplement: S2 Table — (DOCX) [file pone.0261726.s002.docx]

| **Table S2. Adherence with Health Recommendations** | | | | | | |
| --- | --- | --- | --- | --- | --- | --- |
|  | | | | | | |
| **S2_1. Adherence with Health Recommendations of Full Sample** | | | | | | |
|  | Certainly Not | Probably Not | Maybe | Probably Yes | Most Certainly | Missing |
| Washing your hands often | 20 (0.3) | 12 (0.2) | 38 (0.07) | 404 (6.9) | 5347 (91.9) | 66 (1.1) |
| Avoiding close contact with people who are sick | 26 (0.4) | 9 (0.2) | 50 (0.9) | 442 (7.6) | 5274 (90.9) | 86 (1.5) |
| Social isolation even if you have no symptoms (avoiding large crowds) | 38 (0.7) | 44 (0.8) | 143 (2.5) | 845 (14.5) | 4763 (81.7) | 54 (0.9) |
| Avoiding travel | 36 (0.6) | 58 (1.0) | 172 (2.9) | 772 (13.2) | 4802 (82.2) | 47 (0.8) |
| Cough or sneeze into your elbow | 24 (0.4) | 34 (0.6) | 120 (2.1) | 607 (10.4) | 5039 (86.5) | 63 (1.0) |
|  | | | | | | |
| **S2_2. Adherence with Health Recommendations of Purposive Sample** | | | | | | |
|  | Certainly Not | Probably Not | Maybe | Probably Yes | Most Certainly | Missing |
| Washing your hands often | 3 (0.6) | 1 (0.2) | 4 (0.8) | 38 (7.6) | 456 (91.4) | 40 (7.4) |
| Avoiding close contact with people who are sick | 4 (0.8) | 3 (0.6) | 3 (0.6) | 40 (8l.1) | 448 (90.7) | 45 (8.4) |
| Social isolation even if you have no symptoms (avoiding large crowds) | 5 (1.0) | 3 (0.6) | 9 (1.8) | 78 (15.7) | 406 (81.9) | 43 (8.0) |
| Avoiding travel | 6 (1.2) | 8 (1.6) | 11(2.2) | 73 (14.7) | 403 (81.4) | 44 (8.2) |
| Cough or sneeze into your elbow | 2 (0.4) | 0 (0) | 10 (2.0) | 53 (10.6) | 439 (87.5) | 37 (6.9) |
|  | | | | | | |
