# Supplementary material for: ‘Us-Versus-Them’: Othering in COVID-19 public health behavior compliance
Source: PLoS One. 2022 Jan 24;17(1):e0261726. doi: 10.1371/journal.pone.0261726 (PMC8786185; doi:10.1371/journal.pone.0261726)
Supplement: S3 Table — (DOCX) [file pone.0261726.s003.docx]

| **Table S3. Categories and Codes used for subgroup analyses** | |
| --- | --- |
| **Category** | **Codes included in subgroup analysis** |
| Personal Health Concerns | Death of Self |
|  | Fear of Getting Sick |
|  | Inadequate work policies (impact my health) |
|  | Questions or concerns about personal access to care or testing (for self or family) |
|  | Other personal medical concerns |
|  | Family member dying |
|  | Family member illness |
|  | Not being able to provide care for family if sick |
|  | Other family concerns |
|  | At-risk populations (not personal family) |
|  | Concern about mental health |
|  | General loss of life, death (societal level) |
|  | Healthcare workers |
|  | Other safety and health concerns |
| Viral Behavior | Questions or concerns about viral behavior |
| Economic Concerns | Economy crashing |
|  | Job layoffs |
|  | Other economic concerns |
| Vaccine and Medicines | Questions or concerns about medications; drug developments; treatment |
|  | Questions or concerns about lack of vaccines |
| Lack of concern of others | Lack of concern of others |
| Lack of societal preparedness | Questions or concerns about testing |
|  | Lack of societal public health preparedness |
